# Factors Influencing the Willingness of Universities’ Business Management Departments to Implement Online Entrepreneurship Program and Its Effectiveness

**DOI:** 10.3389/fpsyg.2020.00975

**Published:** 2020-06-05

**Authors:** Yu-Min Wang, Chei-Chang Chiou

**Affiliations:** ^1^Department of Information Management, National Chi Nan University, Puli, Taiwan; ^2^Department of Accounting, National Changhua University of Education, Changhua City, Taiwan

**Keywords:** online entrepreneurship, online entrepreneurship program, the effectiveness of online entrepreneurship program, willingness to implement online entrepreneurship program, universities’ business management departments

## Abstract

With the steady increase and popularization of innovations and applications on the Internet, more and more people are searching for and purchasing products online. The boom in e-commerce stimulates opportunities in online entrepreneurship. However, the risks and failure rate for online entrepreneurship are relatively high. Therefore, some universities are standardizing the implementation of online entrepreneurship programs (OEPs) with the aim of equipping students with knowledge for online entrepreneurship through instruction and practical methods to increase the chance of successful online entrepreneurship and also enhance the professional image of the department. The main purpose of the present study is to explore the key influencing factors affecting the willingness of universities’ department of business administration to implement OEP and its effectiveness. Using the technology-organization-environment (TOE) framework by Tornatzky and Fleischer (1990), the Innovation Diffusion Theory (IDT) by Rogers (1983), and the OEP characteristics as the foundation, the present study developed a model to analyze and elucidate the key factors for OEP implementation willingness and OEP effectiveness. Survey data were collected from teachers at universities’ business management departments, and structural equation modeling (smartPLS) was utilized to verify the research model and hypothesis. The present study found that integrating the TOE framework and the IDT can be used to analyze the key factors influencing OEP implementation willingness and its effectiveness at universities’ business management departments. When implementing the implementation of OEP, business management departments at universities need to take into account factors from three contexts: innovation, organization, and environment. Innovative factors greatly influence the willingness of departments to implement OEP, but organizational and environmental factors have a greater influence on the effectiveness of OEP implementation. The results of the present study will enable academia and education practitioners to better understand how to implement OEP and achieve results in the context of business education at universities.

## Introduction

With the globalization connection and popularization of the Internet, coupled with the rapid development of easy-to-use applications and the effective integration of mobile communication devices, the Internet has become an important medium for interacting and communicating with people and organizations and an important technology and transaction platform that is heavily relied upon. As the Internet-based E-commerce market continues to grow and develop, the number of transactions, the amount, and types are constantly increasing, making the online market a place with an extremely high potential for growth and business opportunities.

In recent years, due to the reduced cost of information and communication technology equipment, the rise of open source software, and the continuous development of a large number of fast and easy-to-use building and development tools for web applications, the barrier to entry for online entrepreneurship has been greatly reduced. In addition to the thriving online market, the number of successful cases of online entrepreneurship (e.g., Amazon, Google, Alibaba) continues to increase, attracting many people to invest in online entrepreneurship, and the number of cases of online entrepreneurship is growing rapidly (Matlay and Westhead, 2007; Millman et al., 2009; Pan et al., 2013; Zhang and Zhou, 2015).

Online entrepreneurship refers to an individual or a group of individuals establishing a new business in innovative ways and using the Internet (including wired and wireless Internet) as an operating platform to conduct business operations and related service activities (Millman et al., 2009). Although online entrepreneurship has the advantage of a global market, high profit potential, and a low barrier to entry, entrepreneurs must possess a wide array of knowledge such as information technology and business management. They must also select the correct target market, recruit venture, and working capital, continue to make innovations in services, and keep up with technological advancements (Carmichael, 2013)—these characteristics make entrepreneurship challenging. In addition, factors such as fierce online competition and easy imitability increase the failure rate for online entrepreneurs, who have a success rate of only 2–4% (Ranjan, 2013).

Education is the most direct and effective way to improve the success of entrepreneurship (Rasmussen and Sørheim, 2006; Ranjan, 2013). Many studies indicate that entrepreneurship education allows students to have the necessary knowledge and skills for entrepreneurship and enables them to effectively plan entrepreneurial risks, processes, and activities and to continue through business management and application updates after starting a business (Millman et al., 2009). Through entrepreneurship education programs, students will be more confident, motivated, proactive, and creative (Peterman and Kennedy, 2003; Souitaris et al., 2007; Wu, 2017; Wu and Song, 2019).

Therefore, in the face of the opportunities and challenges of online entrepreneurship, some universities have gradually offered relevant courses, i.e., online entrepreneurship programs (OEPs), that aim to equip students through education and teaching with the skills for online entrepreneurship. Improving opportunities for success in online entrepreneurship not only enhances students’ competitiveness for future employment but can also improve the professional teaching abilities and image of the department. However, the implementation of OEP is an innovation and challenge for many universities. Departments would need to change and adjust the curriculum structure and design, teaching methods, teacher qualifications, and expertise to be oriented toward practice. At the same time, the content of the curriculum should be changed and adjusted according to changes in technology and market applications (Krishnan, 2003; BizMaverick, 2013; Ranjan, 2013; Rasmussen and Sørheim, 2006). In addition, OEPs are still in the development stage, and there is no consistent standard or structure to follow. Therefore, relevant, in-depth research is needed as a reference for universities to implement OEPs.

Applying the technology-organization-environment (TOE) framework (Tornatzky and Fleischer, 1990) and the Innovation Diffusion Theory (IDT) (Rogers, 1983), the present study aims to explore the key factors affecting the willingness to implement OEP and its effectiveness from the organizational level of business management departments at universities. The results of this study provide a practical and academic reference for the field of online entrepreneurship and instruction implementation.

## Literature Review

### Entrepreneurship, Entrepreneurship Education, and OEPs

#### Entrepreneurship and Entrepreneurship Education

Entrepreneurship positively affects a country’s economic development (Oosterbeek et al., 2010; Astebro et al., 2012). When a country has many of its citizens engaging in entrepreneurial activities, new startups bring competition and pressure to existing companies in the industry through the innovative activities and models created by these entrepreneurs and the highly efficient spirit created by active management and investment when starting a business. Existing companies must upgrade their services and products to avoid becoming obsolete. Therefore, entrepreneurship increases the overall economic growth of the country, which is why more and more countries encourage entrepreneurial activities by providing entrepreneurial support and incentives.

Although entrepreneurship benefits a country’s overall development, it is also a high-risk endeavor with a high rate of failure. However, many studies (Lüthje and Franke, 2002; Rasmussen and Sørheim, 2006; Oosterbeek et al., 2010; Ranjan, 2013) indicate that the chance of entrepreneurial success can be increased through effective education and training that has been designed in detail (i.e., entrepreneurship education).

The main purpose of entrepreneurship education is to teach and guide students to effectively apply the theoretical knowledge of textbooks to practice and understand the entrepreneurial spirit; build their entrepreneurial confidence, motivation, and creativity; and transform them into actual entrepreneurial behaviors through practical learning (Laukkanen, 2000; Souitaris et al., 2007; Ranjan, 2013). In terms of teaching methods, entrepreneurship education puts special emphasis on practical learning (i.e., learning by doing) and building knowledge and experience through learning from failure (Oosterbeek et al., 2010).

Different from traditional teaching methods, entrepreneurship education focuses on case teaching to enable students to connect with entrepreneurial ideas and stimulate students’ entrepreneurial spirit in taking risks. As for business ideas, entrepreneurship education focuses on allowing students to seek and discover business opportunities with high potential. Regarding the degree of involvement in entrepreneurial projects, entrepreneurship education encourages active student participation, enabling them to take risks to create a new business. Rasmussen and Sørheim (2006) proposed a figure for analyzing the university strategy for entrepreneurship education. This figure compares traditional education with entrepreneurship education from facets of “focus on business idea” and “student involvement in idea development.” Learning in traditional teaching is more passive and oriented toward individuals while entrepreneur education adopts case-based teaching methods to encourage students to come up with ideas and utilize link analysis on them.

#### Online Entrepreneurship Program

With the popularization of the Internet, the rollout of new innovative applications, and the continuous growth of the e-commerce market, the Internet has become a business market with high potential, and more and more people and businesses look toward the online market with online entrepreneurship intentions. Different from traditional entrepreneurship, online entrepreneurship mainly uses the Internet as the operating platform for business (Wu et al., 2019); at the core of tasks such as profit opportunities and service delivery is the Internet, differing from that of traditional businesses, which emphasizes physical resources such as land, factories, equipment, and materials. In addition, due to the rapid improvements and updates in information and communications technology and the continual decrease in hardware and software costs, the dynamics and variability of online business operations have increased, making the market accessible for competitors, highly competitive, and highly imitable (Millman et al., 2009; Pan et al., 2013; Zhang and Zhou, 2015).

Since there are many types of Internet applications and transactions, academia and the practical world have no consistent and standard way to categorize online entrepreneurship. Batjargal (2007) suggested that there are six types of online entrepreneurship, i.e., Internet service provider, Internet content provider, electronic commerce, networking technology, software development, equipment distributor, and other Internet-related business. BizMaverick (2013) divided online entrepreneurship into five categories: bloggers, affiliate marketers, affiliate website operators, wholesale goods, and any worker who profits online. However, these classification methods do not completely cover online business models. Some classifications overlap, which is why many studies adopted a conceptual definition. Gundry and Kickul (2006), Matlay (2004), and Kollmann (2006) defined online entrepreneurship as a business wholly or partially operating on the Net economy. Manuel (2006) defined online entrepreneurship as establishing business activities on the Internet and selling or providing services as the main means of profiting. Summarizing numerous viewpoints, Millman et al. (2009) defined online entrepreneurship as an individual or a group of individuals partaking in behaviors such as establishing business opportunities, disseminating information, or collaborating with clients and partners through the Internet or mobile technology.

Since online entrepreneurship mainly utilizes the Internet and other related information and communications technology as the operating platform, courses on Internet skills and knowledge must be added to OEP in addition to traditional entrepreneurship courses. Due to the Internet being a virtual world different from the physical world’s business environment, the business conditions, business model, competitive environment, opportunity search, corporate values, transaction security and risk, and consumer behavior all differ from traditional physical businesses. Therefore, the curriculum content and course materials must be adjusted, posing a challenge in innovation for universities intending to implement OEP. Table 1 shows the topics and content of OEPs suggested by many universities and research.

**TABLE 1:** Topics and content of online entrepreneurship programs.

**Researchers/institutes**	**Topics and content**	
E-Community Research Center, 2009	• ICT literacy • Online writing • English competency • Product suitability	• Packaging • Purchase order and delivery • Sustainability of website • E-payment gateway
European Vocational Education Institute, 2020	• Establishment and operating e-commerce • Knowledge of the regulation for e-commerce • Creating business models and business plans • Internet site design and copywriting • Social media in e-business, public relation • Tools of Internet marketing	• Brand creating in e-commerce • Internet advertising/graphics • Selection, analysis of economic data • Market research in internet • Databases, e-business security • Knowledge of specialized computer software
Ranjan, 2013	• Basic required skills: websites and blogs, domain, web hosting, WordPress, Google, search engine, social media	• Important skills: nature and scope of websites, how websites can help readers and Internet users, how websites make money, how to start, run, and maintain a website
Wang et al., 2019	• Leadership facet: effective communication, decision making	
	• Technology utilization facet: programming languages and techniques, file management, computer hardware, multi-media hardware, and website applications	
	• Internet marketing and EC facet: Internet marketing strategies, Pricing strategies, Internet channel strategies, Cost structure analysis, Electronic business models, Resource acquisition, and Cross-border electronic commerce	
Zhang and Zhou, 2015	• Technology facet: fundamental technology, current technology, emerging technology, business-driven technology	
	• Business facet: product opportunity discovery and evaluation, product development and management, marketing, sales and business development, finance and legal issues, leadership and vision	
	• Environment facet: internal environment, external environment	
Waters, 2002	• Required core courses: introduction to electronic business (EB), EB technology, EB customer relationship management, EB-enabled supply chain management, EB enterprise resource planning, EB startup and development, EB practicum	
	• Specialty career tracks: accounting and transaction processing, content creation and management, customer relationship management, EB entrepreneurship and strategy, enterprise integration applications, supply chain management	

### Theoretical Foundation

#### TOE

Tornatzky and Fleischer (1990) proposed the TOE framework, which can be used as the basis for analyzing factors influencing organizations’ decision to adopt an innovation and its implementation effectiveness. The TOE framework argues that innovation adoption by a corporation or organization is always influenced by three main contexts: technology (innovation), organization, and environment. The technological context refers to the functions and benefits that can be created by adopting/using this innovation or technology. The organizational context refers to the relevant conditions and characteristics regarding the organization such as size, formalization, centralization, complexity, human resources, adequacy of resources, availability of specific resources, and innovative attitude of senior management (Tornatzky and Fleischer, 1990; Chau and Tam, 1997; Baker, 2012). The environmental context refers to the external environment in which the company is located, including factors such as industry, industry characteristics, competition in the industry, laws and regulations, the number of service providers, and the government (Tornatzky and Fleischer, 1990).

The TOE framework is a conceptual framework that does not indicate which factors should be included in each context. The important influencing factors are chosen depending on the research subject and innovation type. The main contribution of this framework is to provide researchers with a direction to contemplate and explore influencing factors and it can be integrated with many other innovative or organizational theories to increase the depth of research (Baker, 2012).

Based on the TOE framework as the theoretical foundation, Table 2 shows relevant research regarding organizations’ adoption of innovative technology. Although variables between some studies correspond to slightly different contexts in TOE, the TOE framework has been supported by many empirical studies and can be used to predict, analyze, and explain corporate organizations’ decision-making behaviors to adopt innovative technologies. The TOE framework has been verified by and supported by many innovative technologies (e.g., cross-organizational information systems, knowledge management systems, electronic data exchange, open systems, enterprise systems, RFID), industries (manufacturing, retail, finance, wholesale, health care), and countries (Europe, America, and Asia) (Wang et al., 2010; Baker, 2012; Wang and Wang, 2016).

**TABLE 2:** Prior studies on the TOE framework.

**Research**	**Innovation**	**Predictors**		
		**Technology**	**Organization**	**Environment**
Kuan and Chau, 2001	EDI	Direct benefits, indirect benefits	Cost, technical competences	Industry pressure, government pressure
Lee and Shim, 2007	RFID	Perceived benefits, vendor pressure	Presence of champions, financial resources, technology knowledge	Performance gap, market uncertainty
Mehrtens et al., 2001	Internet	Perceived benefits	Organizational readiness	External pressure
Ramdani et al., 2009	Enterprise system	Relative advantages, compatibility, complexity, trialability, observability	Top management support, organizational readiness, IS experience, size	Industry, market scope, competitive pressure, external IS support
Wang et al., 2010	RFID	Relative advantages, compatibility, complexity	Top management support, size, technology competence	Competitive pressure, trading partner pressure, information density
Wang and Wang, 2016	KMS	Perceived benefits, complexity, compatibility	Sufficient resources, technology competence, top management support, organization culture	Competitive pressure
Zhu et al., 2006	EB	Technology readiness, technology integration	Firm size, global scope, managerial obstacles	Competition intensity, regulatory environment

*EDI, electronic data interchange; RFID, radio frequency identification; KMS: knowledge management system; EB: E-business.*

#### IDT

The IDT is a popular theory used by innovation studies. Proposed by Rogers (1983), the IDT can be used to analyze organizations’ or individuals’ willingness, decision-making behavior, and implementation degree concerning innovation adoption. The IDT argues that the decision of innovation adoption depends on the perceived innovation characteristics of potential adopters. Rogers (1983) identified five important perceived characteristics of innovation. They are relative advantage, compatibility, complexity, trialability, and observability. Moore and Benbasat (1991) not only developed a reliable and valid measure for perceived characteristics of innovation, but also suggested some innovative characteristics such as image, result demonstrability, and voluntariness. Related definitions on each perceived attribute of innovation are shown in Table 3.

**TABLE 3:** Conceptual definitions of the perceived characteristics of innovation.

**Characteristics**	**Conceptual definitions**
Relative advantage	The degree to which an innovation is perceived as better than the idea it supersedes.
Compatibility	The extent to which an innovation is perceived as consistent with the values, experiences, and existing practices of the potential implementers.
Complexity	The degree to which an innovation is perceived as difficult to understand and use.
Trialability	The degree to which an innovation can be experimented with before adoption.
Observability	The degree to which the results of an innovation are observable to others.
Image	The degree to which using an innovation is perceived to help enhance or improve the image or social status of a potential adopter.
Result demonstrability	The tangible results of using an innovation.
Voluntariness	The degree to which use of an innovation is perceived as being voluntary or of free will.

*Data source: Agarwal and Prasad (1998); Rogers (1995); Moore and Benbasat (1991).*

## Research Model and Method

### Research Model and Method

Implementing OEP requires many resources to be invested, new practical designs, innovative teaching methods, and curriculum reform. Therefore, it is an important innovative action for many universities. The TOE framework has been applied to the topic of organizations’ innovation adoption by many empirical studies and has high explanatory power. Therefore, this study intends to use the TOE framework as its theoretical foundation. However, in the TOE framework, T refers to the technological context, i.e., characteristics of innovative technology. Since OEP is not an innovative technology, but an innovative practice, this study replaces the technological context with the innovation context. This study integrates the TOE framework with the IDT as the present study’s foundation. This type of theoretical integration is supported by relevant studies (Bradford and Florin, 2003; Jeyaraj et al., 2006; Nagy, 2010). In addition, this study not only explores the willingness to implement OEP as the dependent variable but also includes the effectiveness of OEP implementation as the dependent variable to increase the depth of the research and obtain more advanced results (Bradford and Florin, 2003; Baker, 2012).

**FIGURE 1:**
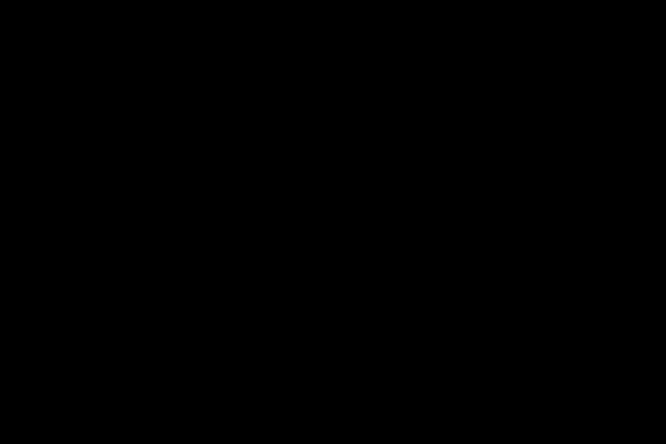
The research model. The only factors significantly affecting departments’ willingness to implement OEP are assumed to be the factors driving the effectiveness.

Integrating the attributes of entrepreneurship programs and online entrepreneurship as well as related literature on organizational innovation adoption, the present study proposes 12 factors from the innovative, organizational, and environmental contexts that may affect the willingness of business management departments at universities to implement OEP and the effectiveness of OEP implementation. The research subjects are the universities’ business management departments. The research model is as shown in Figure 1.

Data collection and analysis were divided into two stages. In the first stage, the present study collected surveys from teachers at universities’ business management departments to obtain key factors affecting the willingness to implement OEP. In the second stage, the subjects of analysis are business management teachers whose departments have implemented OEP, and significant influencing factors analyzed from the first stage were used as the independent variables to verify influencing factors for the effectiveness of implementing OEP.

### Variables Measurement

The measurement of variables in the present study was based on past related literature on organizational innovation adoption and effectiveness, and modified according to the characteristics of entrepreneurship education and online entrepreneurship. All measurement items were measured on a five-point Likert scale (strongly disagree–strongly agree). The contents of the survey were reviewed by nine experts and industry professionals for comprehensiveness, terminology, and relevance. The measurement items for the innovative, organizational, and environmental contexts as well as the dependent variables are as shown in Table 4.

**TABLE 4:** Measurement items.

**Measurement items**
**Relative advantage**
RA1. Compared to the traditional business courses and instruction, OEP will have higher education outcomes.
RA2. Compared to the traditional business courses and instruction, OEP can make students have better business ideas and concepts.
RA3. Compared to the traditional business courses and instruction, OEP can make students have greater entrepreneurial spirit.
RA4. Compared to the traditional business courses and instruction, OEP can make students have higher willingness to try out and take risks.
RA5. Compared to the traditional business courses and instruction, OEP can make students own practical experiences and knowledge.
RA6. Compared to the traditional business courses and instruction, OEP can make students acquire more successful entrepreneurial opportunities.
**Compatibility**
CM1. OEP fits with my department’s teaching practices.
CM2. OEP is consistent with the beliefs and values of my department.
CM3. The implementation of OEP is compatible with the existing instruction infrastructure of my department.
CM4. The implementation of OEP is compatible with the existing instruction resources of my department.
CM5. Attitudes toward OEP in my department have always been favorable.
**Result demonstrability**
RD1. It is not difficult for my department to tell others about the OEP effectiveness.
RD2. It is easy for my department to communicate the OEP consequences with others.
RD3. The results of implementing OEP are apparent to my department.
RD4. It is easy for my department to explain why implementing OEP is beneficial.
**Image**
IM1. Implementing OEP can improve the image of my department.
IM2. If my department implements OEP, others will approve the value of my department.
IM3. Implementing OEP can enhance the prestige of my department.
IM4. Implementing OEP can improve the profile of my department.
IM5. Implementing OEP can improve the academic status of my department.
**Top management support**
MS1. Top management in my university is interested in the implementation of OEP.
MS2. Top management in my university considers implementing OEP to be important.
MS3. Top management in my university supports the implementation of OEP.
**Department size**
DS1. The number of teachers in my department is higher compared to the other related departments.
DS2. The number of students in my department is higher compared to the other related departments.
**Department innovativeness**
DI1. When there are new teaching methods and themes, my department would look for ways to experiment with them.
DI2. Compared to other departments, my department is usually the first to try out teaching inventions.
DI3. My department is unhesitant to try out new teaching methods and themes.
DI4. My department likes to experiment with new teaching methods and themes.
**Department readiness**
DR1. The teachers in my department have related professional knowledge for implementing OEP.
DR2. The administration personnel in my department have related professional knowledge for implementing OEP.
DR3. My department has sufficient financial resources for implementing OEP.
DR4. My department has sufficient external resources for implementing OEP.
DR5. My department can provide students relevant knowledge for starting up a new business.
DR6. My department (university) has good connections with venture investment companies.
DR7. My department (university) can help in the process of starting up a new business.
**Competitive pressure**
CP1. My department experienced competitive pressure to implement OEP.
CP2. Students expect my department can implement OEP.
CP3. My department could have experienced student enrollment pressure.
**External support**
ES1. My department can recruit sufficient qualified professional specialist faculties to participate in OEP.
ES2. My department can invite consultants and professionals with entrepreneurial experiences to participate in OEP.
ES3. My department can invite venture capital firms to participate in OEP.
ES4. My department can invite Internet service providers to participate in OEP.
**Government implementation**
GS1. The government always plays an important role in OEP implementation.
GS2. The government provides sufficient resources to implement university departments to implement OEP.
GS3. The government encourages and supports university departments to implement OEP.
**Implementation willingness**
IW1. My department has the willingness to implement OEP.
IW2. My department will implement OEP in the future.
**Implementation effectiveness**
IE1. The students of my department are willing to start a business online.
IE2. The students of my department are willing to accept the challenge of online entrepreneurship.
IE3. In the past 3 years, the number of online entrepreneurship graduates from my department is higher than that of other departments.
IE4. In the past 3 years, the graduates of my department have a higher number of successful online entrepreneurships than other departments.

## Data Analysis and Hypothesis Verification

### Key Factors Affecting the Willingness to Implement OEP

This study distributed survey questionnaires to teachers responsible for curriculum planning in universities’ business management departments. A total of 105 responses were collected. The sample was 61.9% male, 38.1% female; 27.6% professors, 31.4% associate professors, 34.3% assistant professors, and 6.7% lecturers or others. The higher education system accounted for 56.2%, while the vocational system accounted for 48.3%; 41.9% have already implemented or are planning to implement OEP, while 58.1% are not planning to implement OEP.

The smartPLS software with structural equation modeling was used for data analysis. The analysis procedure and standards were performed according to the recommendations of Hair et al. (2013). After removing items having a factor loading of less than 0.6, the composite reliability (CR) of all variables was greater than 0.6, and all of the average variance extracted (AVE) was greater than 0.5. The square root of the AVE was greater than the correlation coefficient of the two corresponding variables. The results are as shown in Tables 5, 6, which meet the requirements of construct validity.

**TABLE 5:** Composite reliability and AVE (dependent variable: implementation willingness).

**Constructs**	**CR**	**AVE**	**Constructs**	**CR**	**AVE**
Relative advantage	0.90	0.83	Department innovation	0.95	0.84
Compatibility	0.76	0.53	Department readiness	0.94	0.72
Complexity	0.86	0.67	Competitive pressure	0.90	0.82
Result demonstrability	0.90	0.75	External support	0.88	0.65
Image	0.83	0.62	Government support	0.95	0.86
Top management support	0.91	0.76	Implementation willingness	0.95	0.91
Department size	0.92	0.85			

**TABLE 6:** Discriminant validity (dependent variable: implementation willingness).

	**MS**	**RA**	**DI**	**ES**	**IM**	**IW**	**RD**	**GS**	**DR**	**CM**	**CP**	**CX**	**DS**
MS	0.87												
RA	0.08	0.91											
DI	0.58	0.13	0.92										
ES	0.54	0.10	0.63	0.81									
IM	0.49	0.25	0.49	0.38	0.79								
IW	0.61	0.29	0.43	0.52	0.51	0.95							
RD	0.51	0.07	0.43	0.28	0.40	0.55	0.86						
GS	0.51	0.15	0.11	0.53	0.29	0.47	0.22	0.92					
DR	0.58	0.08	0.67	0.68	0.45	0.44	0.14	0.41	0.85				
CM	0.48	0.11	0.50	0.37	0.21	0.35	0.28	0.30	0.69	0.73			
CP	0.57	0.01	0.55	0.53	0.31	0.45	0.44	0.35	0.51	0.32	0.91		
CX	–0.27	–0.08	–0.48	–0.24	–0.35	–0.53	–0.55	–0.08	–0.32	–0.43	–0.30	0.82	
DS	0.06	0.24	0.32	–0.12	0.39	0.15	0.31	–0.23	0.12	–0.17	0.09	–0.16	0.92

*RA, relative advantage; CM, compatibility; CX, complexity; RD, result demonstrability; IM, image; MS, top management support; DS, department size; DI, department innovation; DR, department readiness; CP, competitive pressure; ES, external support; GS, government support; IW, implementation willingness.*

Regarding the significance testing of factors influencing the willingness to implement OEP, this study performed structural equation modeling using smartPLS and set the bootstrapping value at 5000 according to the recommendations of Hair et al. (2013). Due to the exploratory nature of the present study, we extended the significance level to 0.1 as suggested by Hair et al. (2013). There are seven factors significantly affecting the willingness to implement OEP (as shown in Table 7), which are relative advantage, complexity, and image from the innovative context; top management support, department size, and department readiness from the organizational context; and external support from the environmental context. Relative advantage, complexity, and top management support are the top three factors with the most influencing powers.

**TABLE 7:** Testing results of hypotheses affecting implementation willingness.

**Factors**	**β**	**Standard deviation**	***T* value**	***P*-value**
Relative advantage	0.45	0.13	3.39	0.00**
Compatibility	0.32	0.45	0.72	0.47
Complexity	−0.40	0.16	2.55	0.01*
Result demonstrability	0.37	0.35	1.06	0.29
Image	0.26	0.15	1.76	0.08*
Top management support	0.38	0.20	1.91	0.06*
Department size	0.43	0.23	1.84	0.07*
Department innovation	0.19	0.27	0.72	0.48
Department readiness	0.68	0.41	1.66	0.10*
Competitive pressure	0.07	0.13	0.50	0.62
External support	0.50	0.30	1.76	0.08*
Government support	0.21	0.19	1.14	0.26

*P < 0.1, **P < 0.05.

### Key Factors Affecting OEP Implementation Effectiveness

This study distributed survey questionnaires to the teachers responsible for curriculum planning in universities’ business management departments and whose departments had implemented OEP. A total of 65 responses were collected. The sample was 61.5% male, 38.5% female; 24.6% professors, 33.9% associate professors, 32.3% assistant professors, and 9.2% lecturers or others. The higher education system accounted for 56.9%, while the vocational system accounted for 43.1%.

The smartPLS software with structural equation modeling was used for data analysis. The testing results on CR, AVE, and discriminant validity are as shown in Tables 8, 9, which meet the requirements of construct validity.

**TABLE 8:** Composite reliability and AVE (dependent variable: implementation effectiveness).

**Constructs**	**CR**	**AVE**	**Constructs**	**CR**	**AVE**
Relative advantage	0.79	0.67	Department size	0.90	0.82
Complexity	0.84	0.64	Department readiness	0.94	0.72
Image	0.89	0.66	External support	0.92	0.74
Top management support	0.91	0.78	Implementation effectiveness	0.89	0.73

**TABLE 9:** Discriminant validity (dependent variable: implementation effectiveness).

	**MS**	**RA**	**ES**	**IM**	**IE**	**DR**	**CX**	**DS**
MS	0.88							
RA	0.15	0.82						
ES	0.55	0.08	0.86					
IM	0.47	0.08	0.34	0.81				
IE	0.62	0.06	0.60	0.53	0.86			
DR	0.63	0.09	0.74	0.47	0.58	0.85		
CX	–0.40	–0.18	–0.28	–0.41	–0.31	–0.44	0.80	
DS	0.03	0.08	0.13	0.24	0.35	0.15	–0.20	0.90

*RA, relative advantage; CX, complexity; IM, image; MS, top management support; DS, department size; DR, department readiness; ES, external support; IE, implementation effectiveness.*

The smartPLS was used to test hypotheses about factors influencing OPE implementation effectiveness. The bootstrapping value was set to 5000. Due to the exploratory nature of the present study, we extended the significance level to 0.1 as suggested by Hair et al. (2013). There are three factors significantly affecting the willingness to implement OEP (as shown in Table 10), which are top management support, department size, and external support.

**TABLE 10:** Testing results of the hypotheses affecting implementation effectiveness.

**Factors**	**β**	**Standard deviation**	***T* value**	***P*-value**
Relative advantage	0.07	0.13	0.56	0.57
Complexity	0.08	0.11	0.78	0.44
Image	0.18	0.17	1.09	0.28
Top management support	0.33	0.19	1.76	0.08*
Department size	0.41	0.10	4.04	0.00***
Department readiness	−0.12	0.16	0.77	0.44
External support	0.53	0.16	3.26	0.00***

*P < 0.1, ***P < 0.01.

## Discussion

The results of this study showed that integrating the TOE framework and the IDT can effectively analyze the willingness to implement OEP at universities. OEP implementation is not only an issue of educational innovation but also an undertaking of the organization and external environment. As to the effectiveness of OEP implementation, the present study found that the organization and the external environment exert important influences. We discuss each influencing factor in the following passage.

### Innovation Context

The empirical results show that the factors within the innovative context significantly affecting the willingness of universities’ department of business management to implement OEP are relative advantage, complexity, and image. Relative advantage positively affects the willingness of business management departments at universities to implement OEP. Etzkowitz et al. (2000) indicated that OEP implementation can increase students’ practical skills in online entrepreneurship and also enhance the connection between the department and industrial practices, make innovations in teaching methods, strengthen teacher–student network literacy, enhance students’ ultimate learning outcome, and implement universities’ social services and contributions. When departments believe that implementing OEP can improve the effectiveness of education and equip students with better employment competitiveness and advantages, it improves their willingness to implement OEPs.

This study found that complexity negatively affects the willingness of business management departments to implement OEP. Complexity is the perceived difficulty, i.e., the difficulty an organization faces in understanding and learning how to implement an innovation (Rogers, 1983). An innovation with high complexity means that it is hard to understand and to implement, and the risks and uncertainties to implementing this innovation are relatively high. Many studies on organizational innovation (Tornatzky and Klein, 1982; Premkumar et al., 1994) found that complexity is a factor impeding innovation adoption and effectiveness. OEP not only differs from the lectures of the traditional teaching method, but emphasizes case teaching and practical applications. Since online entrepreneurship uses the Internet and other related information and communications technologies as the operating platform, the business conditions, business model, competitive environment, opportunity search, corporate values, transaction security and risk, and consumer behavior all differ from the operations of a physical business. Therefore, when implementing OEP, departments must reform the curriculum content, course materials, and teaching methods, and the course content must be upgraded according to advancements in technology, posing challenges and difficulties to implementing OEPs (Waters, 2002; Gundry and Kickul, 2006; Millman et al., 2009).

This study found that image positively affects the willingness of business management departments to implement OEP. Image refers to the degree to which the organization’s social image or status is enhanced after adopting an innovation. Previous studies on organizational innovation (Karahanna et al., 1999; Carter and Bélanger, 2005) also found that image is a driving factor in innovation adoption and effectiveness. As OEP is in the early stages of popularization, universities implementing OEP will gain a high social evaluation, including teaching methods, teaching innovation, and industrial connections. When the department believes OEP implementation can help gain a better reputation and academic standing, it will increase their willingness to implement OEP.

The empirical results of the present study showed factors within the innovative context such as relative advantage, complexity, and image have no significant influence on the OEP effectiveness. Since these innovation factors are departments’ initial perceived anticipation, actual results are affected by organizational resources, organization support, and physical environment factors.

### Organization Context

The empirical results of this study showed that factors within the organizational context affecting the willingness of universities’ business management departments to implement OEP include top management support, department size, and the department readiness. The influence of top management support on departments’ willingness to implement OEP is very reasonable. To implement a new educational program or change instructional methods, a university-level meeting will review and approve. If top management such as principals or first-level supervisors support the innovation, passing the program is easier. If the opposite occurs, OEP implementation becomes difficult. In addition, to implement OEP, business management departments must acquire funding to obtain relevant resources such as a learning system for experiential simulations of entrepreneurship, entrepreneurs with real entrepreneurial experience, or mentors such as venture capitalists to review whether entrepreneurial undertakings can become a success (Graevenitza et al., 2010; Tian et al., 2016; Troudt et al., 2017). This allows students to enhance their entrepreneurial intentions and cultivate their entrepreneurial abilities, improving their workplace competency and employment rate and giving their departments an incentive to improve OEP (Arpat et al., 2019). Therefore, if top management thinks implementing OEP is important to the university or is interested in and support implementing OEPs, it can increase the willingness of business management departments to implement OEP.

In addition, department size also positively affects business management departments’ willingness to implement OEP. The main reason may be the greater the number of teachers, the more chance some teachers want to actively implement new academic programs. For students of business management, entrepreneurship is one of the important plans for future career developments, and the importance of entrepreneurship education stems from the high youth unemployment rate (Kuratko, 2005; Troudt et al., 2017). Therefore, more and more teachers in business management are encouraging students to start their own business after graduation. In the era of Internet popularization, online entrepreneurship is also a trend. The willingness of business management departments to implement OEP will naturally increase. In particular, departments having more teachers and students will have more incentive to implement OEP.

Another factor within the organizational context positively affecting the willingness of business management departments to implement OEP is department readiness. The more prepared the faculty is for OEP, the greater the willingness to implement OEP will be. Compared to other schools’ business management departments, if teachers and administrators at a school’s business management departments possess relevant resources such as expertise, funding, and an online environment to implement OEP and are equipped to provide students with the knowledge necessary to start a new business, they will be more willing to implement OEPs. Arpat et al. (2019) indicated that the number of youths with entrepreneurial intentions and spirit has exponentially increased, showing that many students intend to start a business and also increasing business management departments’ motivation to implement OEP. However, it is important to effectively construct entrepreneurship education through a holistic perspective and design and implement entrepreneurial course content in a manner ensuring students obtain the necessary qualifications for entrepreneurship. Therefore, business management departments will be more willing to implement OEPs if they are fully prepared and there are a higher number of youths with entrepreneurial or online entrepreneurial intentions.

In terms of the effectiveness of business management departments implementing OEP, top management support and department size are two significant influencing factors. The effect of top management support on OEP effectiveness is higher. We infer if senior management or first-level administrators are interested in or support OEP implementation, they are bound to be more active, giving supporting resources, enriching resources to implementing the program, and ultimately improving its effectiveness. Tian et al. (2016) indicated that the entrepreneurial environment such as education, policies, and funding is an important supporting element to implementing entrepreneurial education for college students. These supporting elements are also the key to the effectiveness of OEP. In addition, department size also positively influences the effectiveness of business management departments’ OEP implementation. We infer that the bigger the department, the more resources will be allocated by the school. This makes resources needed for OEP implementation more abundant, including hiring professionals from practices to co-teach together (Ghina, 2014), holding various entrepreneurial contests (Tian et al., 2016), or simulating entrepreneurship within class so that students have the opportunity to accumulate entrepreneurial experiences during school (Graevenitza et al., 2010; Arpat et al., 2019), increasing their entrepreneurial intentions and enhancing the effectiveness of OEP implementation. A survey by Ghina (2014) also found that one of the causes of students’ poor entrepreneur intention was the lack of standardized methods in entrepreneurship education and in increasing students’ entrepreneurial skills and intentions; the expense of the holistic design in entrepreneurship education must be paid by the department. A large department and great top management support allow more resources and time to achieve a holistic standard and greater effectiveness in implementing entrepreneurship programs.

### Environment Context

The empirical results of this study showed that the main factor within the environmental context affecting the willingness of universities’ business management departments to implement OEP is external support. External support is an important factor for many organizations when evaluating whether to adopt a technological innovation. When introducing innovative technology, the technological skills and service capabilities of related suppliers and whether the company can hire the necessary professionals to introduce innovation all influence decision-making for innovation adoption (Jeyaraj et al., 2006). For OEP, external support includes whether departments can hire sufficient professional teachers from the industry to participate in OEPs, whether they can invite consultants and entrepreneurs with entrepreneurial experience, venture capitalists to participate in OEPs, and companies with online services to participate in OEP. When departments have adequate external support, it naturally has a positive effect on the willingness to implement OEP. Further, the participation of mentors from practices can also increase students’ entrepreneurial motivation and intentions in the future, as well as enhance their understanding of entrepreneurial practices and cultivate their entrepreneurial abilities (Ghina, 2014), which can greatly improve the success rate of OEP implementation and thereby increase business management departments’ willingness to implement OEP.

For factors influencing the effectiveness of business management departments’ OEP implementation, external support from the environmental context positively affects the effectiveness of OEP implementation. This result showed that adequate external support plays a key role in the success of OEP implementation. Tian et al. (2016) indicated that the entrepreneurial environment such as universities’ entrepreneurial education environment and the social and cultural environment is an important external factor for implementing entrepreneurial education for universities.

External support such as inviting mentors like consultants and entrepreneurs, venture capitalists, or online service companies with entrepreneurial experiences to co-sponsor the establishment of OEPs enables students to better understand the practical entrepreneurial skills and professionalism necessary for entrepreneurship in action and make implementing entrepreneurship programs more beneficial. Many teachers at the university do not have entrepreneurial experience; most of the course content is based on theory or books to teach students entrepreneurial knowledge, which may not meet students’ needs or allow students to truly realize the practical aspects of entrepreneurship. Consultants and entrepreneurs, venture capitalists, or online service companies with entrepreneurial experience all have substantial experience in personal entrepreneurship or identifying potential physical or online entrepreneurship businesses. Through sharing actual experiences and student interaction, students can better understand how to start a business, and it can also raise students’ entrepreneurial motivations. Such a pairing of theoretical teaching with practical mentoring is greatly beneficial to students’ absorption of entrepreneurial knowledge and the improvement of their entrepreneurial intentions. A survey by Ghina (2014) found that the reasons for students’ poor entrepreneurial intentions include the lack of ability of teachers to propose new paradigms as to the importance of the entrepreneurial spirit and the lack of proper coordination among universities and entrepreneurs. Aydemir (2018) also indicated that the effective implementation of an entrepreneurial course requires a design in which students can engage in actual entrepreneurial activities. Therefore, providing an ideal learning environment that can simulate entrepreneurship and industry teachers with entrepreneurial experience becomes an indispensable resource (Graevenitza et al., 2010). These soft and physical resources require external support. Therefore, departments must actively attract external support including industry teachers, counseling consultants and venture capitalists with entrepreneurial experience, and vendors of services on online platforms to achieve effective OEP implementation (Rasmussen and Sørheim, 2006; Pan et al., 2013; Wallace, 2013).

## Conclusion

Integrating the TOE framework with the IDT, this study analyzed the key factors influencing OEP implementation at universities’ business management departments and its effectiveness using empirical research methods. We obtained the following four research findings and conclusions:

(1) Integration of the TOE framework and the IDT can be used to analyze key factors influencing OEP implementation at universities’ department of business management and its effectiveness. Business management departments promoting the implementation of OEP must take into account the factors from the three contexts: innovation, organization, and environment.
(2) This study found seven significant factors influencing the willingness to implement OEP: relative advantage, complexity, image, top management support, department size, department readiness, and external support. Relative advantage and complexity are the two most important factors.
(3) For the effectiveness of OEP implementation, this study found three significant factors, which are top management support, department size, and external support.
(4) Innovation factors have a greater influence on business management departments’ willingness to implement OEP, but the organization and the environment factors have greater influences on the effectiveness of OEP implementation. This finding can be used as a reference for departments implementing OEP and for achieving its effects.

## Data Availability Statement

The datasets presented in this article are not readily available because authors have promised not to disclose the survey data of the departments. Requests to access the datasets should be directed to C-CC, ccchiou@cc.ncue.edu.tw.

## Ethics Statement

Ethical review and approval was not required for the study on human participants in accordance with the local legislation and institutional requirements. All respondents filled in the questionnaire from an organizational perspective under their own free will. Written informed consent from the participants was not required to participate in this study in accordance with the national legislation and the institutional requirements.

## Author Contributions

Y-MW contributed to the research topic, research model, data collection, statistical analysis, and writing. C-CC edited this manuscript, developed implications, and responsible for correspondence.

## Conflict of Interest

The authors declare that the research was conducted in the absence of any commercial or financial relationships that could be construed as a potential conflict of interest.
